# Molecular Detection of Bovine Papillomavirus DNA in the Placenta and Blood of Healthy Mares and Respective Foals

**DOI:** 10.3390/vetsci6010014

**Published:** 2019-02-06

**Authors:** Federica Savini, Laura Gallina, Francesca Mazza, Jole Mariella, Carolina Castagnetti, Alessandra Scagliarini

**Affiliations:** Department of Veterinary Medical Sciences, University of Bologna, Via Tolara di Sopra, 50 Ozzano Emilia, 40064 Bologna, Italy; laura.gallina@unibo.it (L.G.); francesca.mazza7@unibo.it (F.M.); jole.mariella2@unibo.it (J.M.); carolina.castagnetti@unibo.it (C.C.); alessand.scagliarini@unibo.it (A.S.)

**Keywords:** bovine papillomavirus, vertical transmission, sarcoid, horse, placenta, veterinary science, infectious diseases

## Abstract

Despite the characteristic species specificity of Papillomaviruses (PVs), the bovine papillomavirus (BPV) types 1, 2, and—more rarely—13, can cross-infect equids, where they are involved in the pathogenesis of sarcoid neoplasms. Sarcoids are locally invasive fibroblastic skin tumors that represent the most common skin neoplasms in horses worldwide. The transmission mechanism of BPV is still controversial in horses. Thus far, direct and indirect routes have been implicated, while vertical transmission has been suggested after the detection of viral DNA in the semen of healthy stallions. Testing of the blood and placenta of non-sarcoid baring mares and their respective foals revealed that the equine placenta can harbor BPV DNA, leading us to speculate a possible prenatal vertical DNA transmission in equids.

## 1. Introduction

Papillomaviruses (PVs) are small double-stranded DNA (dsDNA) viruses capable of infecting all amniotes. Strict species specificity is characteristic of all PVs, however, the bovine papillomavirus (BPV) types 1, 2, and 13 (BPV-1, -2, -13) can cause cross-species infection in equids, where they cause sarcoids [1,2,3]. Sarcoids are locally invasive fibroblastic skin tumors that represent the most common dermatological neoplasm worldwide in horses and can seriously compromise the welfare of affected equids and cause substantial economic losses. BPV DNA has not only been detected in sarcoid lesions but also in the normal skin and blood of horses with and without equine sarcoid [4,5,6,7,8]. Nevertheless, the mode of BPV transmission within equid populations and the presence of latent infections are still controversial: direct and indirect transmission via body contact, contaminated material, and habitual surroundings [4], as well as flies [6], have been implicated. Thus far, vertical transmission has been suggested in equids by evidence of BPV gene expression in the blood and semen of healthy horses [9].

Our study aimed at obtaining information about the possible vertical transmission of BPV DNA from mares to foals, starting with the evidence that BPV DNA has been frequently detected in the blood of healthy horses [6,10]. Studies on the transmission via blood lymphocytes and the placenta are of particular interest due to their implications in the prevention of infection.

## 2. Materials and Methods

Twelve pregnant mares, admitted to the Equine Perinatology Unit of the Department of Veterinary Medical Sciences of the University of Bologna between March 2011 and January 2018, were included in the study (Table 1), based on the absence of clinical signs and anamnestic history of sarcoid lesions but found to be BPV positive in the blood. Immediately after delivery, 3 mL of blood was collected in K_3_EDTA containing tubes both from the mother and from the foal before suckling. The placenta was also sampled, avoiding environmental contamination, and immediately stored at −70 °C. The peripheral blood mononuclear cells (PBMCs) were separated with Ficoll^®^ from whole blood, frozen, and kept at −70 °C. DNA was extracted from the placental tissue and PBMCs with a commercial kit (MN, NucleoSpin Tissue, Macherey Nagel, Düren, Germany). Parallel, BPV DNA negative skin biopsies obtained from sheep were extracted as a negative control. In order to check the DNA integrity, the horse β-actin housekeeping gene was amplified in each sample. PCR with consensus primers (5′B1/2-L1: GCTAAGCAACAACAGATTCTGTTGC; 3′B1/2-L1: TCAGCCATTTTGAGGTAGTCTGG) targeting a 266 bp region of the major capsid gene L1 of BPV-1 and -2 [11] were used to determine the presence of viral DNA. Amplicons of positive samples were sequenced using both forward and reverse primer on an ABI Prism 3100 Genetic Analyzer (Perkin-Elmer Applied Biosystems, Foster City, CA, USA).

## 3. Results

Positivity to amplification with the primers for the horse β-actin gene demonstrated the viability of the genomic DNA in all the tested samples. As expected, no amplification was obtained from the skin biopsies of the sheep, while BPV DNA was amplified from the placenta, PBMCs of mares, and PBMCs of respective foals in seven out of twelve couples.

Alignment of the sequenced amplicons showed the presence of a different percentage of identity (Table 1). Within couples, 100% of nucleotide identity among the three matrices was identified only in one case (couple N. 4), while in the remaining cases, the identity percentage between maternal blood and respective foal varied between 93.8% and 98.8%. In four cases, the sequence obtained from the placenta showed 100% identity to the sequence obtained from the foal blood sample (N. 1, 3, 6, and 7). The results obtained from the BLAST^®^ for each sample suggested the presence of viral DNA referable to BPV-1, BPV-2, and BPV-1 EqSarc.

## 4. Discussion

Different tissues and fluids, such as peripheral blood [12,13], blood plasma [14], milk [15], ovaries, uterus, semen, and spermatozoa [16,17,18] were found positive for BPV-1/-2 DNA in cattle. In equids, DNA and gene expression of BPV have been demonstrated in the peripheral blood and skin of sarcoid and non-sarcoid baring horses, showing that the virus can cause asymptomatic infections, with BPV remaining latent in its episomal form [18,19]. Trans-placental infection of the fetus is not without controversy, but HPV DNA has also been identified in the amniotic fluid [20], placenta, and umbilical cord [21], leading to speculate that infection might take place during pregnancy in the female [22]. In addition, vertical transmission has been documented in cattle [14,16,17,23] and water buffalo [24]. In our study, the detection of BPV DNA in the placenta and blood of newborn foals suggested that the peripheral blood of the mother could be argued as being a vehicle of viral DNA dissemination. The presence of BPV DNA in the blood and placenta could provide hints for the vertical transmission of the virus, although clinical consequences need to be further investigated. Vertical transmission has also been recently suggested in sheep, another Delta-BPV cross-infected species [25]. The authors reported the detection of Bovine δPV-2 and δPV-13 in the congenital neoplastic lesions of lambs and in the blood of their mothers, pointing out that haematogenous transplacental transmission can occur also in sheep.

Sequencing results showed a high genetic heterogeneity among the three matrices within couples. In particular, the lack of sequence concordance between the maternal blood and placenta revealed a high degree of L1 genetic variability as already shown for the BPV E5 gene in the blood of healthy horses [10]. Since only a small segment of the genome was sequenced, it was not possible to classify the BPV type with confidence, but the sequence alignments suggested the presence of both BPV-1 and BPV-2 even in different matrices within the same couple. In analogy to our findings, non-concordance of the viral type has been described for HPV infection in women [26,27,28] and has been justified by a previous vertical transmission at other times during pregnancy when maternal sampling was not performed. In fact, as we collected maternal samples for BPV DNA detection at only one point in time during pregnancy, we may have missed the critical maternal infection responsible for the vertical transmission. Additionally, Sanger sequencing lacks resolution when compared to next-generation sequencing (NGS), since only the major variant driving the infection can be reliably determined [29]. In this context, it could be assumed that the mare may be infected by different viral variants or types, and either only one was able to trans pass the placental barrier or all types can do it but only one can be detected. Nevertheless, infection could be also ascribable to the oocyte fecundation, as already suggested in humans [30] since gene expression of E5 BPV has been detected in the semen of healthy stallions [9]. Prophylactic implications could also be considered when subjects are intended for reproduction; in fact, even though the significance of BPV DNA in healthy horses has not been elucidated yet, trophoblasts are the target of HPV and BPV infections in women and cattle respectively [31,32]. Taken all together, our data illustrated the need for a better understanding of the clinical significance of BPV DNA in non-epithelial tissues of horses and, in particular, in healthy mares, where implications in reproduction have never been considered.

## Figures and Tables

**Table 1:** Sample sequencing results and percent identity within couples. The table shows the nucleotide percent identities of the sequences obtained from maternal blood (M) compared to the placenta (P) and foal blood (F) sequences respectively. In brackets are reported the BPV types (GenBank: BPV-1 X02346; BPV-1 EqSarc JX678969.1; BPV-2 M20219) with the best identity score retrieved from BLAST^®^ database for each sample’s sequence.

Couple N°	Maternal Blood ID (BLAST^®^ Identity)	Placenta ID (BLAST^®^ Identity)	Foal Blood ID (BLAST^®^ Identity)
**1**		184 P (99.5% BPV-1)	185 F (100% BPV-1 EqSarc)
	184 M (99.1% BPV-2)	95%	95%
**2**		461 P (100% BPV-1 EqSarc)	462 F (99.1% BPV-2)
	461 M (99.5% BPV-1 EqSarc)	100%	93,8%
**3**		695 P (100% BPV-1)	696 F (100% BPV-1)
	695 M (96.6% BPV-1 EqSarc)	95%	95%
**4**		620 P (100% BPV-1 EqSarc)	621 F (100% BPV-1 EqSarc)
	620 M (99.5% BPV-1 EqSarc)	100%	100%
**5**		623 P (99.5% BPV-2)	622 F (98.7% BPV-2)
	623 M (99.5% BPV-1 EqSarc)	95%	93.8%
**6**		633 P (100% BPV-1)	634 F (99.5% BPV-1)
	633 M (98.7% BPV-2)	93.8%	93.8%
**7**		246 P (100% BPV-1 EqSarc)	276 F (99.5% BPV-1 EqSarc)
	246 M (99.1% BPV-1 EqSarc)	98.8%	98.8%

## References

[1] Chambers G., Ellsmore V.A., O’Brien P.M., Reid S.W.J., Love S., Campo M.S., Nasir L. (2003). Association of bovine papillomavirus with the equine sarcoid. J. Gen. Virol..

[2] Lunardi M., de Alcântara B.K., Otonel R.A., Rodrigues W.B., Alfieri A.F., Alfieri A.A. (2013). Bovine papillomavirus type 13 DNA in equine sarcoids. J. Clin. Microbiol..

[3] Van Dyk E., Oosthuizen M.C., Bosman A.M., Nel P.J., Zimmerman D., Venter E.H. (2009). Detection of bovine papillomavirus DNA in sarcoid-affected and healthy free-roaming zebra (Equus zebra) populations in South Africa. J. Virol. Methods.

[4] Bogaert L., Martens A., De Baere C., Gasthuys F. (2005). Detection of bovine papillomavirus DNA on the normal skin and in the habitual surroundings of horses with and without equine sarcoids. Res. Vet. Sci..

[5] Bogaert L., Martens A., Van Poucke M., Ducatelle R., De Cock H., Dewulf J., De Baere C., Peelman L., Gasthuys F. (2008). High prevalence of bovine papillomaviral DNA in the normal skin of equine sarcoid-affected and healthy horses. Vet. Mic..

[6] Brandt S., Haralambus R., Schoster A., Kirnbauer R., Stanek C. (2008). Peripheral blood mononuclear cells represent a reservoir of bovine papillomavirus DNA in sarcoid-affected equines. J. Gen. Virol..

[7] Finlay M., Yuan Z., Burden F., Trawford A., Morgan I.M., Campo M.S., Nasir L. (2009). The detection of Bovine Papillomavirus type 1 DNA in flies. Virus Res..

[8] Gaynor A.M., Zhu K.W., Dela Cruz F.N., Affolter V.K., Pesavento P.A. (2016). Localization of Bovine Papillomavirus Nucleic Acid in Equine Sarcoids. Vet. Pathol..

[9] Silva M.A., Silva K.M., Jesus A.L., Barros L.O., Corteggio A., Altamura G., Borzacchiello G., Freitas A.C. (2014). The presence and gene expression of bovine papillomavirus in the peripheral blood and semen of healthy horses. Transbound. Emerg. Dis..

[10] Savini F., Gallina L., Prosperi A., Battilani M., Bettini G., Scagliarini A. (2015). E5 nucleotide polymorphisms suggest quasispecies occurrence in BPV-1 sub-clinically infected horses. Res. Vet. Sci..

[11] Brandt S., Haralambus R., Shafti-Keramat S., Steinborn R., Stanek C., Kirnbauer R. (2008). A Subset of Equine Sarcoids Harbours BPV-1 DNA in a Complex with L1 Major Capsid Protein. Virology.

[12] Campo M.S., Jarrett W.F.H., O’Neil B.W., Barron R.J. (1994). Latent papillomavirus infection in cattle. Res. Vet. Sci..

[13] Roperto S., Comazzi S., Ciusani E., Paolini F., Borzacchiello G., Esposito I., Roperto F. (2011). PBMCs are additional sites of productive infection of bovine papillomavirus type 2. J. Gen. Vir..

[14] Freitas A.C., Silva M.A.R., Carvalho C.C.R., Birgel E.H., dos Santos J.F., Beçak W., Stocco dos Santos R.C., Méndez-Vilas A., Formatex B. (2007). Papillomavirus DNA detection in non epithelial tissues: A discussion about bovine papillomavirus. Communicating Current Research and Educational Topics and Trends in Applied Microbiology.

[15] Lindsey C.L., Almeida M.E., Vicari C.F., Carvalho C., Yaguiu A., Freitas A.C., Beçak W., Stocco R.C. (2009). Bovine papillomavirus DNA in milk, blood, urine, semen, and spermatozoa of bovine papillomavirus-infected animals. Genet. Mol. Res..

[16] Yaguiu A., De Carvalho C., de Freitas A.C., Luiz G. (2006). Papillomatosis in cattle: In situ detection of bovine papillomavirus DNA sequences in reproductive tissues. Braz. J. Morphol. Sci..

[17] De Carvalho C., de Freitas A.C., Brunner O., Bentim Góes L.G., Yaguiu Cavalcante A., Beçak W., Stocco dos Santos R.C. (2003). Bovine papillomavirus type 2 in reproductive tract and gamets of slaughtered bovine females. Braz. J. Microbiol..

[18] Silva M.A., Pontes N.E., Da Silva K.M., Guerra M.M., Freitas A.C. (2011). Detection of bovine papillomavirus type 2 DNA in commercial frozen semen of bulls (Bos taurus). Anim. Reprod. Sci..

[19] Carr E.A., Theon A.P., Madewell B.R., Griffey S.M., Hitchcock M.E. (2001). Bovine papillomavirus DNA in neoplastic and nonneoplastic tissues obtained from horses with and without sarcoids in the western United States. Am. J. Vet. Res..

[20] Martens A., de Moor A., Demeulemeester J., Peelman L. (2003). Polymerase chain reaction analysis of the surgical margins of equine sarcoids for bovine papilloma virus DNA. J. Gen. Virol..

[21] Armbruster-Moraes E., Ioshimoto L.M., Leão E., Zugaib M. (1994). Presence of Human Papillomavirus DNA in Amniotic Fluids of Pregnant Women with Cervical Lesions. Gynecol. Oncol..

[22] Rombaldi R.L., Serafini E.P., Mandelli J., Zimmermann E., Losquiavo K.P. (2009). Perinatal transmission of human papilomavirus DNA. Virol. J..

[23] Stocco dos Santos R.C., Lindsey C.J., Ferraz O.P., Pinto J.R., Mirandola R.S., Benesi F.J., Birgel E.H., Pereira C.A., Beça W. (1998). Bovine papillomavirus transmission and chromosomal aberrations: An experimental model. J. Gen. Virol..

[24] Russo V., Paciello O., Zicarelli G., Urraro C., Ceccarelli D.M., De Falco F., Roperto S. (2017). Placental papillomatosis in water buffalo associated with bovine deltapapillomavirus. Proc. LXXI Soc. Vet. Sci..

[25] Roperto S., Russo V., Corrado F., Munday J.S., De Falco F., Roperto F. (2018). Detection of bovine Deltapapillomavirus DNA in peripheral blood of healthy sheep (Ovis aries). Transbound. Emerg. Dis..

[26] Sarkola M.E., Grénman S.E., Rintala M.A., Syrjänen K.J., Syrjänen S.M. (2008). Human Papillomavirus in the Placenta and Umbilical Cord Blood. Acta Obstet. Gynecol. Scand..

[27] Smith E.M., Parker M.A., Rubenstein L.M., Haugen T.H., Hamsikova E., Turek L.P. (2010). Evidence for Vertical Transmission of HPV from Mothers to Infants. Infect. Dis. Obstet. Gynecol..

[28] Lee S.M., Park J.S., Norwitz E.R., Koo J.N., Oh I.H., Park J.W., Kim S.M., Kim Y.H., Park C.W., Song Y.S. (2013). Risk of Vertical Transmission of Human Papillomavirus throughout Pregnancy: A Prospective Study. PLoS ONE.

[29] Shen-Gunther J., Wang Y., Lai Z., Poage G.M., Perez L., Huang T.H. (2017). Deep sequencing of HPV E6/E7 genes reveals loss of genotypic diversity and gain of clonal dominance in high-grade intraepithelial lesions of the cervix. BMC Genom..

[30] Foresta C., Patassini C., Bertoldo A., Menegazzo M., Francavilla F., Barzon L., Ferlin A. (2011). Mechanism of human papillomavirus binding to human spermatozoa and fertilizing ability of infected spermatozoa. PLoS ONE.

[31] Hermonat P.L., Kechelava S., Lowery C.L., Korourian S. (1998). Trophoblasts are the preferential target for human papilloma virus infection in spontaneously aborted products of conception. Hum. Pathol..

[32] Roperto S., Borzacchiello G., Esposito I., Riccardi M., Urraro C., Lucà R., Corteggio A., Tatè R., Cermola M., Paciello O. (2012). Productive infection of bovine papillomavirus type 2 in the placenta of pregnant cows affected with urinary bladder tumors. PLoS ONE.

